# SLE-Associated rs2295613(A) Allele Strengthens a Predicted c-MYC Motif and Enhances *SLAMF1* Promoter Reporter Activity in B Cells

**DOI:** 10.3390/cells15171568

**Published:** 2026-08-28

**Authors:** Aksinya N. Uvarova, Lidia V. Putlyaeva, Kirill V. Korneev, Ekaterina M. Stasevich, Elvina A. Prikhodko, Matvey M. Murashko, Denis E. Demin, Elina A. Zheremyan, Anton M. Schwartz, Dmitry V. Kuprash

**Affiliations:** 1 Engelhardt Institute of Molecular Biology, Russian Academy of Sciences, 119991 Moscow, Russiakuprash@gmail.com (D.V.K.); 2 Center for Regenerative Medicine, Medical Research and Educational Institute, Lomonosov Moscow State University, 119234 Moscow, Russia; 3 School of Medicine, University College Dublin, D04 V1W8 Dublin, Ireland; 4 Conway Institute for Biomolecular and Biomedical Research, University College Dublin, D04 V1W8 Dublin, Ireland; 5 Department of Human Biology, Faculty of Natural Sciences, University of Haifa, Haifa 3498838, Israel

**Keywords:** *SLAMF1*, CD150, SLE, B cells, c-MYC, rs2295613, rs2295614, SNP, allele-specific regulation

## Abstract

**Highlights:**

**What are the main findings?**

- rs2295613(A) increased *SLAMF1* promoter reporter activity in Raji, MP1, and primary CD19^+^ B cells.
- Motif disruption and partial MYC depletion reduced the activity of the rs2295613(A)-containing reporter.

**What are the implications of the main findings?**

- The regulatory activity associated with rs2295613(A) was reproducible across transformed and primary B-cell reporter systems, although its effect at the endogenous *SLAMF1* locus remains to be established.
- The contrasting reporter effects observed across B- and T-cell models indicate that the regulatory consequence of rs2295613 depends on the cellular transcriptional environment.

**Abstract:**

*SLAMF1* encodes CD150, an immunoregulatory receptor involved in lymphocyte activation, T–B-cell interactions, and humoral immune responses. The *SLAMF1* promoter polymorphism rs2295613(G>A) was previously associated with systemic lupus erythematosus (SLE) susceptibility in a Chinese case–control cohort. Here, we investigated the regulatory activity of rs2295613 in the transformed B-cell lines Raji and MP1 and in primary human CD19^+^ B cells. The rs2295613(A)-containing reporter showed higher promoter activity than the rs2295613(G)-containing reporter in all three cellular systems. Bioinformatic analysis predicted that the G to A substitution strengthens a pre-existing MYC-compatible motif. Substitutions disrupting the motif-containing region attenuated the rs2295613(A)-associated increase in reporter activity and reduced enrichment of the promoter fragment in anti-c-MYC DNA pull-down assays. Partial siRNA-mediated reduction in MYC mRNA also decreased the activity of the rs2295613(A)-containing reporter in Raji cells. Together, these findings identify rs2295613 as a functional *SLAMF1* promoter variant in B-cell reporter systems and support a contribution of c-MYC-associated regulation to the enhanced activity of the rs2295613(A)-containing promoter.

## 1. Introduction

Systemic lupus erythematosus (SLE) is a chronic autoimmune inflammatory disease characterized by impaired immune tolerance, dysregulated B-cell activation, autoantibody production, and abnormalities in T-cell-mediated immune regulation, including altered T-helper and T-regulatory lymphocyte function [1,2]. Genetic susceptibility to SLE is shaped by multiple coding and non-coding variants. A substantial fraction of SLE-associated polymorphisms is located outside protein-coding regions and may influence disease risk by altering promoter or enhancer activity, transcription factor binding, chromatin state, or inducible gene expression in specific immune cell types [3,4]. The SLAM/CD2 gene cluster was implicated in lupus susceptibility through characterization of the murine Sle1b locus, where extensive polymorphisms were identified in genes encoding signaling lymphocytic activation molecule family receptors [5]. SLAM family receptors are broadly expressed on hematopoietic cells and regulate lymphocyte activation, adhesion, and intercellular signaling. In particular, SLAMF1, SLAMF5, and SLAMF6 contribute to the regulation of humoral immune responses [6]. *SLAMF1* encodes signaling lymphocytic activation molecule family member 1, also known as CD150. SLAMF1 mediates homophilic interactions between immune cells, including T–B-cell contacts, and participates in lymphocyte activation and immune synapse regulation. Increased SLAMF1 expression has been reported on helper T cells and B cells from patients with active SLE [7]. Single-cell mass cytometry analysis further showed that SLAMF1-expressing B cells are increased in SLE and that SLAMF receptor co-expression marks SLE-associated immune signatures, including switch memory B-cell and circulating T follicular helper cell populations [8]. Functionally, SLAMF1 engagement can inhibit T–B-cell interaction and reduce IL-6 production and plasmablast differentiation in SLE-derived cellular systems [9]. These data support the relevance of SLAMF1 expression changes for immune-cell interactions involved in SLE pathogenesis.

Two single-nucleotide polymorphisms located in the *SLAMF1* promoter, rs2295614(A>T) and rs2295613(G>A), were previously examined in a Chinese SLE case–control cohort [10]. Increased susceptibility was associated with the rs2295614(A)–rs2295613(G) haplotype, whereas rs2295613(A) was less frequent among patients and occurred in the reduced-risk rs2295614(T)–rs2295613(A) haplotype. Because the rs2295614(A)–rs2295613(A) haplotype was not associated with SLE, the independent contribution of rs2295613 remained unresolved. This prompted us to examine the regulatory effect of rs2295613 on both rs2295614 backgrounds. The frequency of rs2295613(A) also varies substantially among ancestry groups, being markedly higher in East Asian populations than in the other major 1000 Genomes populations (Table S1). In a previous reporter study, rs2295613(A) showed different effects in two transformed T-cell models, increasing *SLAMF1* promoter activity in MT-2 cells and decreasing it in Jurkat cells, whereas rs2295614(T) showed no detectable independent effect [11]. These observations indicated that the reporter effect of rs2295613(A) is cell-context-dependent but did not establish how the variant acts in B cells. Bioinformatic analysis identified an E-box-like sequence overlapping rs2295613 that is compatible with MYC/MAX recognition. Because c-MYC participates in B-cell proliferation, metabolism, and germinal-center responses [12,13,14], this sequence represented a candidate contributor to the rs2295613(A)-associated reporter effect.

Here, we examined the reporter activity of *SLAMF1* promoter constructs carrying rs2295613(G) or rs2295613(A) in the transformed B-cell lines Raji and MP1 and in primary human CD19^+^ B cells. Constructs representing all four rs2295614–rs2295613 combinations were additionally analyzed in Raji cells to determine whether the reporter effect of rs2295613 depended on the rs2295614 background. To assess the contribution of the local MYC-compatible sequence, we combined targeted sequence disruption, siRNA-mediated reduction in MYC mRNA in Raji cells, and DNA pull-down followed by anti-c-MYC immunoprecipitation. This design allowed us to test whether rs2295613(A) increases *SLAMF1* promoter reporter activity in B-cell systems and whether this effect is associated with the integrity of the predicted local MYC-compatible sequence.

## 2. Materials and Methods

### 2.1. Cell Culture

The EBV-positive B-lymphoblastoid cell line MP1 [15] was kindly provided by Dr. Edward A. Clark (University of Washington, Seattle, WA, USA), and the EBV-positive Burkitt’s lymphoma cell line Raji [16] was kindly provided by Dr. Sergey E. Dmitriev (Belozersky Institute of Physico-Chemical Biology, Lomonosov Moscow State University, Moscow, Russia). Primary human B cells were isolated from the peripheral blood of three healthy adult donors, who provided written informed consent. Donors were not genotyped at rs2295613 or rs2295614. Peripheral blood mononuclear cells (PBMCs) were isolated by Ficoll-Hypaque (1.077 g/cm^3^, PanEco, Moscow, Russia) density gradient centrifugation (400× *g*, 30 min). CD19^+^ cells were isolated from PBMCs using a human CD19^+^ MACS Cell Isolation Kit (Miltenyi Biotec, Bergisch Gladbach, Germany). All cells were cultivated in RPMI 1640 medium (PanEco, Moscow, Russia) supplemented with 10% FBS (Corning, New York, NY, USA), 2 mM L-glutamine, 1 mM sodium pyruvate, 100 U/mL penicillin, 100 μg/mL streptomycin (all PanEco, Moscow, Russia), non-essential amino acids, and 10 mM HEPES (all Gibco, Bleiswijk, The Netherlands).

### 2.2. Reporter Plasmids, Cell Transfection and Luciferase Reporter Assay

Firefly luciferase reporter constructs containing the *SLAMF1* promoter region chr1:160616732–160617376, hg19, previously characterized by Schwartz et al. [17], were generated in our earlier study of *SLAMF1* promoter polymorphisms [11]. Briefly, the promoter fragment was amplified from genomic DNA and cloned upstream of the firefly luciferase gene into the pGL3-basic vector (Promega, Madison, WI, USA). The minor rs2295613(A) and rs2295614(T) variants were introduced by PCR-directed mutagenesis using overlapping primers: F-rs2295613(A), 5′-GGGGCACATGTTTCTGCC-3′; R-rs2295613(A), 5′-AACATGTGCCCCAGCTTCTTT-3′; F-rs2295614(T), 5′-CAAGAAGTCTTACCGGAGAGAA-3′; R-rs2295614(T), 5′-CGGTAAGACTTCTTGGATTGCAA-3′. An additional rs2295613(A)-based construct carrying targeted point mutations that disrupt the predicted c-MYC site was generated by PCR-directed mutagenesis using overlapping primers: F-rs2295613-dis-mut, 5′-GGGAACAAATTTCTGCCC-3′; R-rs2295613-dis-mut, 5′-AAATTTGTTCCCCAGCTTCT-3′. The double-minor rs2295614(T)–rs2295613(A) promoter construct was generated by overlap-extension PCR from two partially overlapping *SLAMF1* promoter fragments using the internal primers overlap_SLAMF1-R, 5′-AGCTGTCTCCAGGAACCGGAT-3′, and overlap_SLAMF1-F, 5′-ATCCGGTTCCTGGAGACAGCT-3′. The overlapping fragments were combined in an overlap PCR to generate the full-length promoter insert, which was subsequently cloned into the pGL3-basic vector. Plasmid DNA was isolated using the Plasmid Midiprep kit (Evrogen, Moscow, Russia) and verified by Sanger sequencing (EIMB RAS “Genome” center, Moscow, Russia). For the luciferase assay, 2.5 × 10^6^ cells were transfected with 5 μg of test plasmids combined with 0.5 μg of pRL-CMV Renilla luciferase control vector from the Dual-Luciferase Reporter Assay System (Promega, Madison, WI, USA). Cells were electroporated using the Neon Transfection System (Invitrogen, Carlsbad, CA, USA) with appropriate parameters: one 30 ms, 1300 V pulse for Raji cells; three 10 ms, 1300 V pulses for MP1; and one 20 ms, 1900 V pulse for CD19^+^ primary cells. Luciferase activity was measured 24 h post-transfection using a 20/20n Luminometer (Turner BioSystems, Sunnyvale, CA, USA).

### 2.3. siRNA-Mediated c-MYC Knockdown, RNA Extraction, cDNA Synthesis and Quantitative Real-Time PCR

For c-MYC perturbation experiments, siRNA-mediated c-MYC knockdown was performed as described previously for Raji cells [18] using the following c-MYC-targeting siRNA oligonucleotides reported by Reyes-González et al. [19]: F-siRNA-c-MYC, 5′-GCUUGUACCUGCAGGAUCUdTdT-3′; R-siRNA-c-MYC, 5′-AGAUCCUGCAGGUACAAGCdTdT-3′. Scrambled control RNA oligonucleotides were designed using InvivoGen siRNA Wizard v3.1: F-siRNA-scr, 5′-GAGGCGUUUCACGACUUCUdTdT-3′; R-siRNA-scr, 5′-AGAAGUCGUGAAACGCCUCdTdT-3′. RNA extraction, cDNA synthesis, and quantitative real-time PCR were performed as described previously [20] using the following primers: F-RT-c-MYC, 5′-GGACCCGCTTCTCTGAAAGG-3′; R-RT-c-MYC, 5′-CTAACGTTGAGGGGCATCGT-3′; F-B-actin, 5′-TGCGTGACATTAAGGAGAAG-3′; R-B-actin, 5′-GTCAGGCAGCTCGTAGCTCT-3′, using the Applied Biosystems 7500 Real-Time PCR System and the qPCRmix-HS SYBR + LowROX kit (Evrogen, Moscow, Russia).

### 2.4. DNA Pull-Down Assay

Nuclear extracts were prepared from Raji and MP1 cells by nonionic-detergent lysis followed by high-salt extraction. Briefly, 3 × 10^7^ cells were collected by centrifugation at 1200× *g* for 5 min at 4 °C, washed with ice-cold PBS, and resuspended at 1 × 10^7^ cells/mL in buffer A containing 0.32 M sucrose, 3 mM CaCl_2_, 2 mM magnesium acetate, 0.1 mM EDTA, 10 mM Tris-HCl (pH 8.0), 1 mM DTT, 0.5 mM PMSF, 0.5% NP-40, and SIGMAFAST protease inhibitor cocktail (S8820; Sigma-Aldrich, Burlington, MA, USA). The suspension was vortexed for 10 s and incubated on ice for 5 min. Nuclei were collected by centrifugation at 13,200× *g* for 15 s at 4 °C and resuspended in an equal volume of low-salt buffer B containing 20 mM HEPES (pH 7.9), 25% glycerol, 1.5 mM MgCl_2_, 20 mM KCl, 0.2 mM EDTA, 0.5 mM DTT, 0.5 mM PMSF, and protease inhibitors. High-salt buffer C, containing 20 mM HEPES (pH 7.9), 25% glycerol, 1.5 mM MgCl_2_, 0.8 M KCl, 0.2 mM EDTA, 1% NP-40, 0.5 mM DTT, 0.5 mM PMSF, and protease inhibitors, was then added at a 2:1 volume ratio of nuclear suspension to buffer C. After incubation on ice for 15 min, the samples were centrifuged at 13,200× *g* for 20 min at 4 °C. The supernatants containing nuclear proteins were aliquoted and stored in liquid nitrogen. Protein concentration was determined using the Bradford assay. The DNA pull-down assay was performed according to the general procedure described previously [20], using DNA fragments and antibodies specific to the present study. A single primer pair was used to generate 171-bp *SLAMF1* promoter amplicons containing rs2295613(G), rs2295613(A), or the disrupted (A) sequence: F-PD-rs2295613, 5′-ATCCAAGAAGACTTACCGGAGA-3′, and R-PD-rs2295613, 5′-CAAGAAGCCAGGCACTTAGC-3′. Plasmids carrying the corresponding promoter sequences were used as PCR templates. A 196-bp negative-control fragment (chr20:44,746,637–44,746,832, hg19), lacking both a canonical E-box (CACGTG) and a high-confidence predicted MYC motif, was amplified from human genomic DNA (Promega, Madison, WI, USA) using F-PD-control, 5′-ATGGGATCTCTCTCCGGTCG-3′, and R-PD-control, 5′-ATTCACCGCGCAGGAGTTTC-3′. For each binding reaction, 100 ng of one *SLAMF1* promoter amplicon and 100 ng of the negative-control fragment were incubated with 50 µg of nuclear-extract protein in a final volume of 100 µL. The binding buffer contained 60 mM KCl, 12 mM HEPES (pH 8.0), 4 mM Tris-HCl (pH 8.0), 0.5 mM EDTA, 5% glycerol, 1 mM DTT, SIGMAFAST protease inhibitors, and 5 µg of single-stranded salmon sperm DNA as a nonspecific competitor. After incubation on ice for 1 h, 2 µg of anti-c-MYC rabbit monoclonal IgG (ab32072; ChIP grade; Abcam, Cambridge, UK) or 2 µg of an irrelevant isotype-matched rabbit monoclonal IgG against Securin/PTTG1, a nuclear protein not known to associate with the *SLAMF1* promoter region (ab79546; Abcam, Cambridge, UK), was added. Following an additional 1-h incubation on ice, DNA–protein–antibody complexes were captured using Protein A Mag Sepharose, washed, eluted, and neutralized.

DNA present in the precipitates was quantified by real-time PCR. The assay readout was expressed as relative amplicon enrichment rather than as a percentage of input. For each promoter amplicon, the promoter-to-negative-control DNA ratio in the anti-c-MYC precipitate was divided by the corresponding ratio in the anti-Securin/PTTG1 precipitate. The assay was performed in three independent experiments.

### 2.5. Bioinformatic and Statistical Analysis

**Figure 1:**
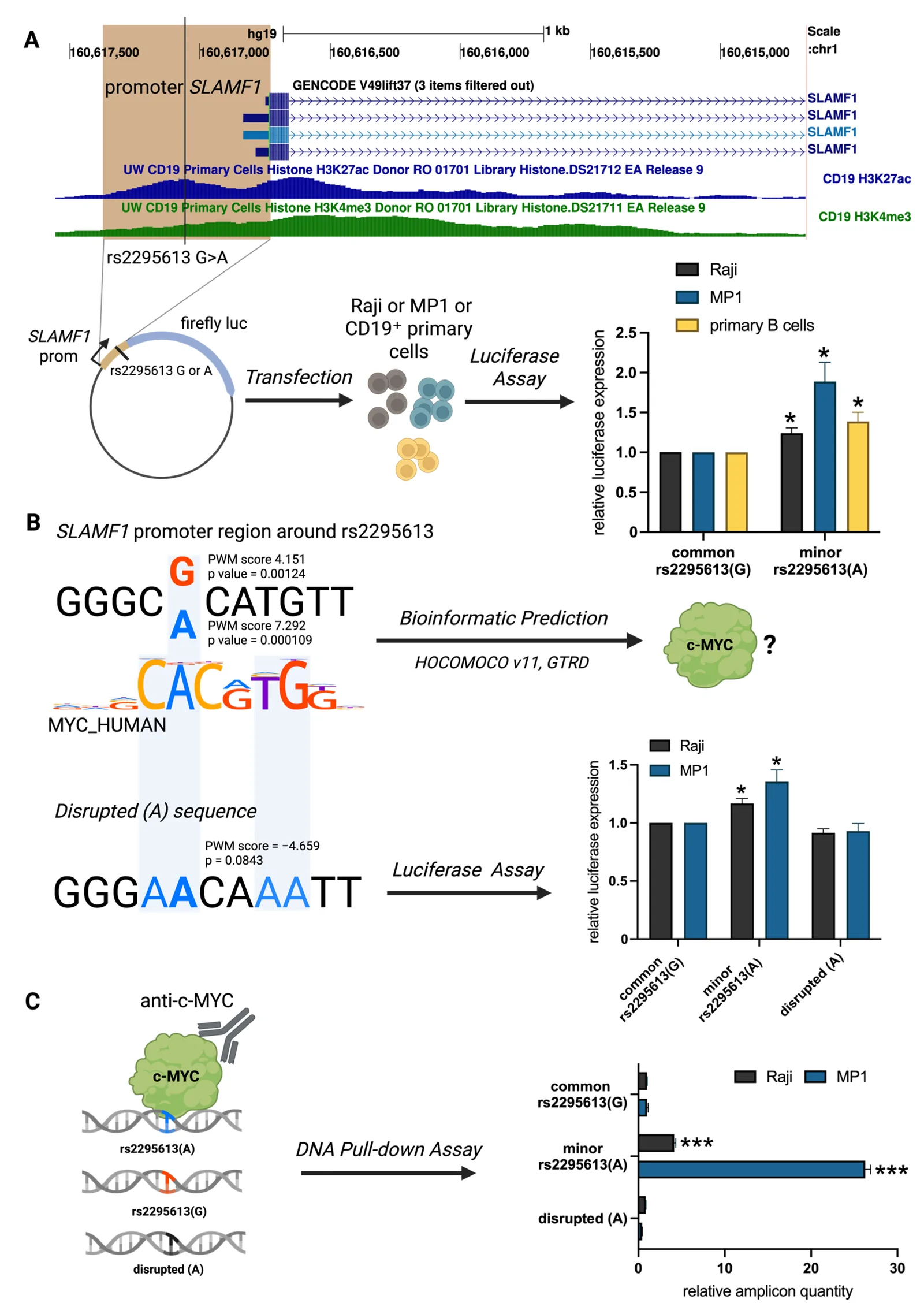
Functional analysis of rs2295613-dependent *SLAMF1* promoter activity and its association with c-MYC-containing protein complexes. (**A**) Genomic location of rs2295613 within the *SLAMF1* promoter region, visualized using the UCSC Genome Browser (GRCh37/hg19). The promoter fragment used in the reporter constructs is shaded in beige. H3K27ac and H3K4me3 ChIP-seq signals in primary human CD19^+^ cells are shown. The schematic illustrates transfection of *SLAMF1* promoter reporter constructs carrying rs2295613(G) or rs2295613(A). Reporter activity was measured in Raji cells (*n* = 9), MP1 cells (*n* = 13), and primary human CD19^+^ B cells (*n* = 3). Firefly luciferase activity was normalized to Renilla luciferase activity and then expressed relative to the rs2295613(G) construct. Data are shown as mean ± SEM. Statistical comparisons were performed on paired Firefly/Renilla ratios before normalization of the rs2295613(G) construct to 1. * *p* < 0.05, two-tailed paired Student’s *t*-test. (**B**) PERFECTOS-APE analysis of the rs2295613-containing sequence using the HOCOMOCO v11 MYC model MYC_HUMAN.H11MO.0.A. The sequence is shown in the orientation of the cloned *SLAMF1* promoter; the predicted MYC-compatible motif is located on the opposite strand. The PWM scores and exact motif-match *p*-values were 4.151 and 0.00124 for rs2295613(G), 7.292 and 0.000109 for rs2295613(A), and −4.659 and 0.0843 for disrupted (A), respectively. Reporter activities of the rs2295613(G), rs2295613(A), and disrupted (A) constructs were measured in Raji (*n* = 4) and MP1 (*n* = 6) cells. Firefly luciferase activity was normalized to Renilla luciferase activity and then expressed relative to the rs2295613(G) construct. Data are shown as mean ± SEM. Statistical comparisons were performed on paired Firefly/Renilla ratios before normalization of the rs2295613(G) construct to 1. * *p* < 0.05, two-tailed paired Student’s *t*-test. (**C**) DNA pull-down analysis of the association between rs2295613-containing *SLAMF1* promoter fragments and c-MYC-containing protein complexes. Amplicons carrying rs2295613(G), rs2295613(A), or disrupted (A) were incubated with nuclear extracts from Raji or MP1 cells and precipitated using an anti-c-MYC antibody or an irrelevant rabbit monoclonal antibody as a control. Amplicon enrichment in the precipitates was quantified by real-time PCR and normalized first to the negative-control DNA fragment and then to the corresponding amplicon precipitated with the irrelevant antibody. Data from three independent experiments are shown as mean ± SEM. Asterisks above the rs2295613(A) bars indicate significant enrichment relative to both the rs2295613(G) and disrupted (A) fragments within the same cell line. *** *p* < 0.001, two-tailed unpaired Student’s *t*-test. Schematics were created with BioRender.com.

The predicted MYC-compatible motif overlapping rs2295613 was analyzed using PERFECTOS-APE/MACRO-APE v3.0.6 [21] and the HOCOMOCO v11 human CORE model MYC_HUMAN.H11MO.0.A [22]. The rs2295613(G), rs2295613(A), and disrupted (A) sequences were analyzed on both DNA strands using identical parameters. The promoter sequence is shown in the cloned orientation in Figure 1B; the overlapping MYC motif match was identified on the complementary strand and is displayed in reverse-complement orientation. PWM scores and exact motif-match *p* values were determined for the same aligned site in all three sequences. The rs2295613-containing *SLAMF1* promoter region was also examined for MYC ChIP-seq evidence in GTRD [23], which identifies the overlapping binding site ID 6481688, located at chr1:160617008–160617060 (hg19).

Statistical analysis was performed using GraphPad Prism 10.6.1 (GraphPad Software Inc., San Diego, CA, USA). Paired two-tailed Student’s *t*-tests were used for the within-experiment reporter comparisons shown in Figure 1A,B and Figure S1A–F,H. The *MYC* mRNA comparison shown in Figure S1G and the DNA pull-down comparisons shown in Figure 1C were analyzed using two-tailed unpaired Student’s *t*-tests. Technical replicate measurements were averaged within each independent experiment before statistical analysis. Each resulting experiment-level value was treated as one observation. Here, *n* denotes the number of independent experiments; for primary CD19^+^ B cells, *n* denotes the number of independent donors. Data are presented as mean ± SEM. No adjustment for multiple comparisons was applied. Exact *p* values for the comparisons presented in Figure S1 are provided in the corresponding panels; asterisks in Figure 1 denote significance thresholds as defined in the figure legend. Differences were considered statistically significant at *p* < 0.05.

## 3. Results

### 3.1. rs2295613(A) Increases SLAMF1 Promoter Activity in B-Cell Lines and Primary Human B Cells

To determine whether rs2295613 affects *SLAMF1* promoter activity in B-cell systems, we compared luciferase reporter constructs carrying rs2295613(G) or rs2295613(A) on the rs2295614(A) background. The constructs were transfected into the transformed B-cell lines Raji and MP1 and into primary human CD19^+^ B cells, and promoter activity was measured using a dual-luciferase reporter assay. In all three cellular systems, the rs2295613(A)-containing construct showed higher reporter activity than the corresponding rs2295613(G)-containing construct (Figure 1A and Figure S1A–C). Because the genetic association of rs2295613 was originally identified within rs2295614–rs2295613 haplotypes, we additionally examined all four allelic combinations in Raji cells. The rs2295613(A)-associated increase in reporter activity was observed on both rs2295614 backgrounds, whereas rs2295614(T) alone did not produce a comparable effect (Figure S1D). Thus, in the tested B-cell reporter systems, increased promoter activity was consistently associated with rs2295613(A), including in primary human B cells.

### 3.2. MYC-Associated Regulation Contributes to the Enhanced Promoter Activity of the rs2295613(A)-Containing Construct

Bioinformatic analysis indicated that rs2295613 lies within a sequence compatible with a MYC binding motif. Targeted analysis with PERFECTOS-APE using the HOCOMOCO v11 human MYC model MYC_HUMAN.H11MO.0.A showed that the rs2295613(G)- and rs2295613(A)-containing sequences had PWM scores of 4.151 and 7.292, respectively, with corresponding exact motif-match *p*-values of 0.00124 and 0.000109. Thus, the G to A substitution strengthened a pre-existing predicted MYC-compatible sequence. The same site-matched analysis of the disrupted (A) construct yielded a PWM score of −4.659 and a motif-match *p*-value of 0.0843, confirming that the three introduced substitutions markedly weakened the predicted match to the MYC model (Figure 1B). The rs2295613-containing region also overlapped a GTRD MYC binding region (Site ID 6481688) supported by MYC ChIP-seq data, including evidence obtained in the Raji Burkitt lymphoma B-cell line. These observations provided additional support for investigating a possible contribution of MYC-associated regulation at this promoter region. To assess the functional importance of the local sequence, we generated a reporter construct carrying rs2295613(A) together with three substitutions within the predicted motif-containing region—disrupted (A). In both Raji and MP1 cells, the disrupted (A) construct showed lower reporter activity than the rs2295613(A) construct, reaching a level comparable to that of the rs2295613(G) construct (Figure 1B and Figure S1E,F). These results indicate that the enhanced reporter activity associated with rs2295613(A) depends on the integrity of the surrounding local sequence.

As an additional functional test, we partially reduced *MYC* expression in Raji cells using a MYC-targeting siRNA. The siRNA decreased *MYC* mRNA expression relative to the scrambled siRNA control (Figure S1G). Under control conditions, the rs2295613(A)-containing reporter showed higher activity than the rs2295613(G)-containing reporter (Figure S1H). Partial reduction in MYC expression decreased the activity of the rs2295613(A)-containing reporter. Together, the effects of disrupting the local MYC-compatible sequence and reducing *MYC* expression support a contribution of c-MYC-associated regulation to the enhanced reporter activity associated with rs2295613(A).

### 3.3. The rs2295613(A)-Containing Promoter Fragment Shows Preferential Enrichment in Anti-c-MYC DNA Pull-Down Assays

To examine whether the rs2295613 alleles differ in their association with c-MYC-containing protein complexes, we performed DNA pull-down assays using promoter amplicons carrying rs2295613(G), rs2295613(A), or disrupted (A). The amplicons were incubated with nuclear extracts from Raji or MP1 cells; DNA–protein complexes were precipitated using an anti-c-MYC antibody or an irrelevant isotype-matched antibody control, and the DNA was quantified by real-time PCR. The rs2295613(A)-containing promoter fragment showed greater relative enrichment than the rs2295613(G)-containing fragment in both cell lines, with stronger enrichment observed in MP1 nuclear extracts (Figure 1C). The disrupted (A) fragment showed markedly lower relative enrichment than the rs2295613(A)-containing fragment, reaching a level close to that of rs2295613(G). These data support greater association of c-MYC-containing protein complexes with the rs2295613(A)-containing *SLAMF1* promoter sequence. The reduced enrichment of disrupted (A) further indicates that this association depends on the integrity of the local motif-containing sequence.

## 4. Discussion

The present study shows that the rs2295613(A)-containing *SLAMF1* promoter reporter has higher activity than the corresponding rs2295613(G)-containing reporter in the transformed B-cell lines Raji and MP1 and in primary human CD19^+^ B cells. Analysis of all four rs2295614–rs2295613 combinations in Raji cells further showed that the rs2295613(A)-associated increase was detectable on both rs2295614 backgrounds, whereas rs2295614(T) alone did not produce a comparable effect. Thus, among the two promoter variants examined, rs2295613 showed the most consistently detectable effect across the tested B-cell reporter systems. These functional results do not resolve the independent genetic contribution of rs2295613 to SLE susceptibility, but they show that its reporter effect is reproducible in several B-cell contexts and is not restricted to a single transformed cell line.

Several observations support a contribution of c-MYC-associated regulation to this reporter effect. PERFECTOS-APE analysis using the HOCOMOCO v11 MYC model showed that rs2295613(A) strengthens a pre-existing predicted MYC-compatible sequence. The rs2295613-containing region also overlaps a GTRD-annotated MYC binding region supported by ChIP-seq evidence, including data obtained in Raji cells. Three substitutions introduced into the motif-containing region markedly weakened the predicted MYC match and reduced the activity of the rs2295613(A)-containing reporter in both Raji and MP1 cells. In addition, siRNA-mediated reduction in MYC mRNA decreased the activity of the rs2295613(A)-containing reporter in Raji cells. Finally, the rs2295613(A)-containing promoter fragment showed greater enrichment in the anti-c-MYC DNA pull-down assay than the rs2295613(G)-containing fragment, whereas the disrupted (A) fragment showed reduced enrichment. These findings support an association between the rs2295613(A) reporter effect and c-MYC-containing protein complexes.

However, the motif-disrupting substitutions alter the local DNA sequence and may also affect the binding of other DNA-binding proteins, local DNA shape, or other sequence-dependent properties. Similarly, the anti-c-MYC DNA pull-down assay compares the relative enrichment of promoter fragments in material precipitated with an anti-c-MYC antibody and does not demonstrate direct binding of c-MYC to the rs2295613-containing sequence. The siRNA experiment provides an additional functional connection to *MYC*, although reduced MYC abundance may influence reporter activity through both direct and indirect transcriptional effects. The combined evidence therefore supports a contribution of c-MYC-associated regulation without establishing specificity for c-MYC or direct c-MYC binding.

The increased reporter activity observed in the B-cell systems is consistent with our previous results in MT-2 cells but differs from the decreased activity observed in Jurkat cells [11]. This divergence among cellular models suggests that the direction and magnitude of the rs2295613(A)-associated reporter effect depend on the cellular transcriptional environment. You et al. [10] examined these promoter variants at the haplotype level, whereas our matched-construct comparisons were designed to isolate the reporter contribution of rs2295613 in B-cell models. Factors such as the abundance and activity of MYC-associated complexes, cooperation with other transcription factors, and the activation state of the cells may influence the direction and magnitude of the reporter response. Previous work in human B-cell lines identified EBF1 as an important regulator of *SLAMF1* promoter activity [17]. That study characterized the broader transcriptional regulation of the promoter, whereas our results add evidence of a local allele-sensitive regulatory effect associated with rs2295613. Together, these findings indicate that the rs2295613-dependent effect occurs within a broader B-cell transcriptional regulatory framework. The present experiments were not designed to determine which additional factors account for the differences among cellular models.

The functional reporter results should also be distinguished from the previously reported population association. In the Chinese case–control cohort, rs2295613(A) was less frequent in patients and was present in the reduced-risk rs2295614(T)–rs2295613(A) haplotype, whereas the rs2295614(A)–rs2295613(A) haplotype was not associated with SLE [10]. Therefore, a protective effect cannot be assigned to rs2295613(A) independently of its haplotypic background. The present study addresses the regulatory properties of rs2295613-containing promoter constructs and does not establish that rs2295613 independently affects SLE susceptibility.

The study has several limitations. Although the reporter effect was reproduced in primary CD19^+^ B cells, this analysis included only three donors, who were not genotyped for rs2295613 or rs2295614. The paired comparison of the two reporter constructs within each donor evaluates the regulatory difference between the cloned promoter sequences but does not address genotype-dependent regulation of endogenous *SLAMF1*. Raji and MP1 are transformed B-cell lines, and episomal reporter constructs do not reproduce the endogenous chromatin environment of the *SLAMF1* locus. The study also did not examine endogenous *SLAMF1* mRNA or surface CD150 expression, and reduction in MYC was confirmed at the mRNA level without a corresponding measurement of c-MYC protein. Further studies will be required to determine whether rs2295613 affects endogenous *SLAMF1* regulation and to establish the molecular composition of the complexes associated with this promoter region.

## 5. Conclusions

The rs2295613(A)-containing *SLAMF1* promoter reporter was more active than the rs2295613(G)-containing reporter in Raji and MP1 cells and in primary human CD19^+^ B cells. Motif-disrupting substitutions, partial reduction in MYC mRNA, and greater relative enrichment of the rs2295613(A)-containing fragment in material precipitated with anti-c-MYC antibodies support a contribution of c-MYC-associated regulation to this reporter effect. Direct c-MYC binding, regulation of endogenous *SLAMF1*, and relevance to SLE susceptibility remain to be established.

## Data Availability

The original contributions presented in this study are included in the article/Supplementary Materials. Further inquiries can be directed to the corresponding author.

## Supplementary Materials

The following supporting information can be downloaded at: https://www.mdpi.com/article/10.3390/cells15171568/s1, Figure S1: Individual experimental values for *SLAMF1* promoter reporter assays and siRNA-mediated reduction in MYC mRNA; Table S1: Allele frequencies of rs2295613 in 1000 Genomes reference populations and in a Chinese SLE case–control cohort.
