# Supplementary material for: SLE-Associated rs2295613(A) Allele Strengthens a Predicted c-MYC Motif and Enhances SLAMF1 Promoter Reporter Activity in B Cells
Source: Cells. 2026 Aug 28;15(17):1568. doi: 10.3390/cells15171568 (PMC13565327; doi:10.3390/cells15171568)
Supplement: Supplementary file 1 [file cells-15-01568-s001.zip › cells-4470762-supplementary.pdf]

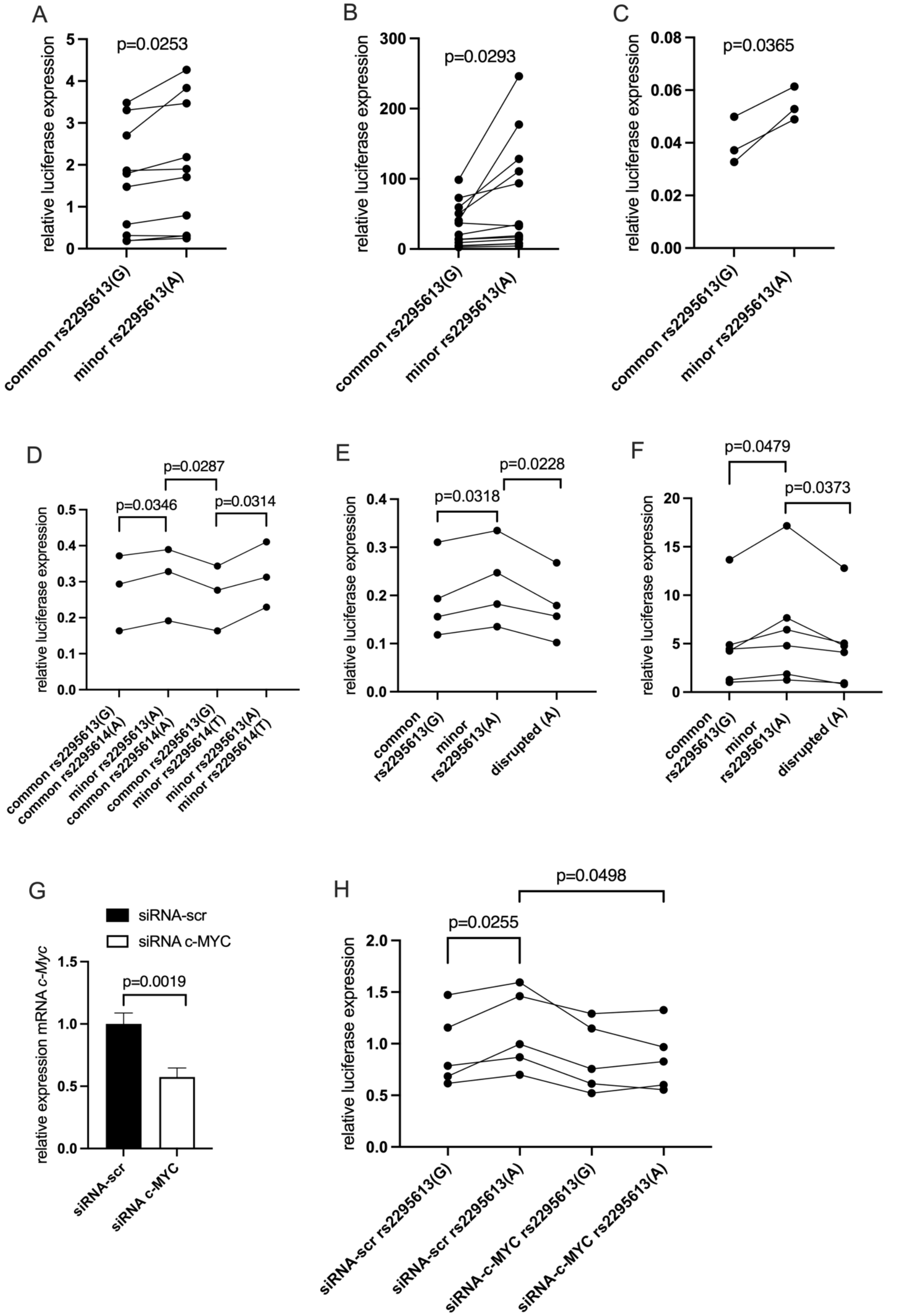

Figure S1. Individual experimental values for *SLAMF1* promoter reporter assays and siRNA-mediated reduction in MYC mRNA **(A–C)** Paired firefly/Renilla luciferase ratios obtained for *SLAMF1* promoter constructs carrying rs2295613(G) or rs2295613(A) in Raji cells (n = 9), MP1 cells (n = 13), and primary human CD19[+] B cells (n = 3),

respectively. **(D)** Reporter activity of constructs representing the four rs2295614–rs2295613 combinations in Raji cells: rs2295614(A)/rs2295613(G), rs2295614(A)/rs2295613(A), rs2295614(T)/rs2295613(G), and rs2295614(T)/rs2295613(A) (n = 3). **(E, F)** Reporter activity of constructs carrying rs2295613(G), rs2295613(A), or rs2295613(A) together with three substitutions within the predicted MYC-compatible motif-containing sequence (disrupted-A) in Raji (n = 4) and MP1 (n = 6) cells, respectively. **(G)** Relative *MYC* mRNA expression in Raji cells transfected with scrambled control siRNA or MYC-targeting siRNA (n = 4). *MYC* mRNA expression was normalized to *ACTB* and expressed relative to the scrambled siRNA control. Bars represent mean ± SEM. **(H)** Reporter activity of the rs2295613(G)- and rs2295613(A)-containing constructs in Raji cells following transfection with scrambled control or MYC-targeting siRNA (n = 5). Firefly luciferase activity was normalized to Renilla luciferase activity. In panels A–F and H, each line connects values obtained within the same independent experiment. The indicated comparisons in panels A–F and H were performed using two-tailed paired Student's t-tests; the comparison in panel G was performed using a two-tailed unpaired Student's t-test. Exact p values are shown in the corresponding panels. Here, n denotes the number of independent experiments; for primary $CD19^+$ B cells, n denotes independent donors.

Supplementary Table S1. Allele frequencies of rs2295613 in 1000 Genomes reference populations and in a Chinese SLE case–control cohort.

Dataset	Population or cohort	Code	rs2295613(A), %	rs2295613(G), %
**1000 Genomes Project**	All populations	ALL	14.10	85.90
	African	AFR	8.77	91.23
	Admixed American	AMR	9.08	90.92
	East Asian	EAS	36.61	63.39
	European	EUR	7.36	92.64
	South Asian	SAS	8.59	91.41
	Han Chinese in Beijing	CHB	34.47	65.53
	Southern Han Chinese	CHS	34.76	65.24
**Chinese SLE case-control cohort** (reported by You et al.)	Patients with SLE	-	35.80	64.20
	Healthy controls	-	45.40	54.60

Notes. Values are allele frequencies. The Chinese case–control frequencies are presented descriptively as reported by You et al. [10]. In that study, rs2295614 and rs2295613 were designated −262A/T and −188A/G, respectively. Because the reported SLE associations depended on the rs2295614–rs2295613 haplotypic background, these frequencies should not be interpreted as evidence that rs2295613(A) independently confers protection against SLE. The 1000 Genomes and Chinese case–control estimates were obtained from independent samples and were not compared statistically. Ensembl reports rs2295613 as C/T on the genomic strand. For consistency with the promoter-oriented constructs used in this manuscript, the allele labels were converted without changing the reported frequencies: genomic T corresponds to promoter A, and genomic C corresponds to promoter G.

Sources. Population allele frequencies for rs2295613 were obtained through the Ensembl REST Variation endpoint for *Homo sapiens* using the 1000 Genomes Project Phase 3 dataset (1000GENOMES:phase_3; accessed on 3 August 2026). Allele frequencies for the Chinese SLE case–control cohort were taken from You et al. [10]: *Detection and functional evaluation of −262A/T and −188A/G polymorphisms of SLAM gene in patients with systemic lupus erythematosus*. *J. Rheumatol.* 2010, **37**, 2268–2272. https://doi.org/10.3899/jrheum.091390.

Abbreviations. AFR, African; AMR, Admixed American; CHB, Han Chinese in Beijing; CHS, Southern Han Chinese; EAS, East Asian; EUR, European; SAS, South Asian; SLE, systemic lupus erythematosus.
